# Sequential Utilization of Hosts from Different Fly Families by Genetically Distinct, Sympatric Populations within the *Entomophthora muscae* Species Complex

**DOI:** 10.1371/journal.pone.0071168

**Published:** 2013-08-08

**Authors:** Andrii P. Gryganskyi, Richard A. Humber, Jason E. Stajich, Bradley Mullens, Iryna M. Anishchenko, Rytas Vilgalys

**Affiliations:** 1 Department of Biology, Duke University, Durham, North Carolina, United States of America; 2 USDA-ARS BioIPM Research, RW Holley Center for Agriculture and Health, Ithaca, New York, United States of America; 3 Department of Plant Pathology and Microbiology, University of California Riverside, Riverside, California, United States of America; 4 Department of Entomology, University of California Riverside, Riverside, California, United States of America; 5 M. Kholodny Institute of Botany, National Academy of Sciences of Ukraine, Kyiv, Ukraine; Wadsworth Center, United States of America

## Abstract

The fungus *Entomophthora muscae* (*Entomophthoromycota*, *Entomophthorales*, *Entomophthoraceae*) is a widespread insect pathogen responsible for fatal epizootic events in many dipteran fly hosts. During epizootics in 2011 and 2012 in Durham, North Carolina, we observed a transition of fungal infections from one host, the plant-feeding fly *Delia radicum,* to a second host, the predatory fly *Coenosia tigrina*. Infections first appeared on *Delia* in the middle of March, but by the end of May, *Coenosia* comprised 100% of infected hosts. Multilocus sequence typing revealed that *E. muscae* in Durham comprises two distinct subpopulations (clades) with several haplotypes in each. Fungi from either clade are able to infect both fly species, but vary in their infection phenologies and host-specificities. Individuals of the more phylogenetically diverse clade I predominated during the beginning of the spring epizootic, infecting mostly phytophagous *Delia* flies. Clade II dominated in late April and May and affected mostly predatory *Coenosia* flies. Analysis of population structure revealed two subpopulations within *E. muscae* with limited gene exchange. This study provides the first evidence of recombination and population structure within the *E. muscae* species complex, and illustrates the complexity of insect-fungus relationships that should be considered for development of biological control methods.

## Introduction

*Entomophthora muscae* (Cohn) Fresen. is the type and the first-described species of the phylum *Entomophthoromycota* Humber [1], [2]. This species kills primarily muscoid flies, and is mostly known from temperate zones of Europe and North America [3], [4]. The number of species in the genus *Entomophthora* has not been completely determined, and *E. muscae* can be considered to be a species complex consisting of several genetically distinct species that are difficult to distinguish morphologically [3], [5]. This species infects hosts from a range of fly families: Anthomyiidae, Calliphoridae, Drosophilidae, Empididae, Muscidae (the most typical hosts), Sarcophagidae and Syrphidae [6]. The *E. muscae* species complex has been split into four groups based on the number and size of conidial nuclei, size of conidia, and host affinity; this has resulted in the recognition of several species in this species complex including *E. scatophagae* and *E. schizophorae* [4], [7]–[9]. Despite a very wide potential host range for some lineages in the *E. muscae* species complex, its constituent taxa do show some host specificities as indicated by decreased cross-infection efficiency between representatives of even a single fly family [10]–[13].

The initial aim of our study was to describe the epidemiology of *E. muscae* infections of fly populations in Durham, NC and to determine the taxonomic identity, host range and any unique ecological, morphological and genetic characteristics of the pathogen. Our investigation revealed that two different fly species were infected by *E. muscae* in the same habitat during each of two consecutive infection seasons. Hypotheses tested were: H_1_ a host switch occurred by the population of the pathogen or H_2_ two different fungal populations infecting different hosts co-exist.

## Results

### Environmental Observations

**Figure 1:**
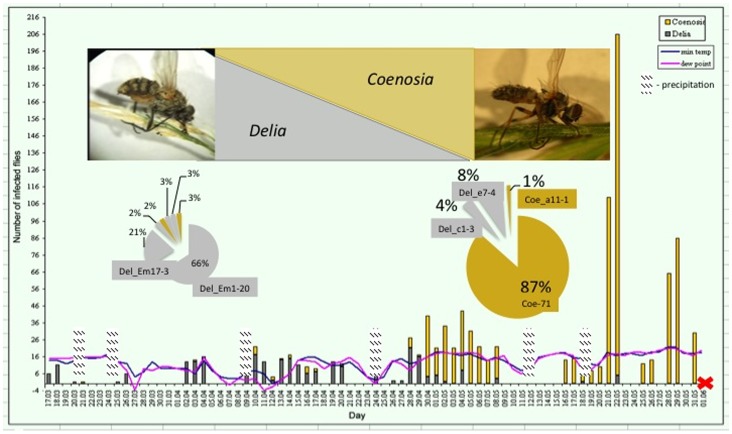
Dynamics of fly infection occurrence, March-May 2012. Red cross marks the end of the epizootic. Circles show the percentage of ITS haplotypes in the beginning (left) and the end of spring (right), their diameters are proportional to the number of presented samples. Del_ indicate fungal sequences extracted from *Delia* (grey rectangles), Coe_ – from *Coenosia* (yellowish rectangles).

Between 3 and 200 infected flies were collected per day in Durham, North Carolina from plants on open urban lawns during the period of 17 March to the end of May 2011–2012. No infected flies were found throughout summer or autumn during weekly surveys. All dead flies were collected in the morning from the tops of plants, usually at a height of 10–70 cm above ground level. Typically, fly cadavers occurred singly on plants. Infected flies were firmly attached to plants by the proboscis and usually with the legs embracing the stalk (**Figs. 1** **,** **2** **A, B, D**). Flies were located close to the apices of plants, with their wings outspread; the head lowered towards the substrate and were vertically oriented with the head either up or down. The abdomen was usually raised. This body position, as well as the localization of the fly to the top of the plant, are hypothesized to increase the effectiveness of conidia discharge by the fungus [14].

**Figure 2:**
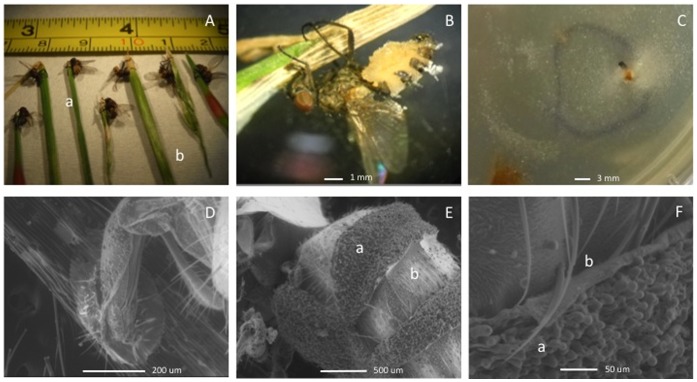
*Entomophthora*-infected *Delia* flies from Durham, North Carolina, USA. A. Fly cadavers on wild onions (a) and grasses (b). B. Fresh fly firmly attached to wild onion; the yellow color of the abdomen indicates active sporulating of conidia. C. Sporulation from an infected fly. D. Proboscis of the fly attached to the plant. E. Fly abdomen, sporulating with conidia (a) on comparatively young “hymenial” zones separated by dorsal cuticular tergites (b). F. Conidiophores (a) breaking through mechanically folded cuticle (b).

In March and the first half of April in both years, about 70% of the infected flies, all of which belonged to the genus *Delia*, were observed on wild onion (or other tall growing plants on local lawns in early spring) [15]. Later in the season, infected specimens of both *Delia* and *Coenosia* were found mostly on wild grasses. The range of environmental conditions favorable for occurrence of dead (presumably infected) flies included average daily temperatures of 11–28°C and relative humidities of 36–87%, according to local weather report data [16], [17]. Other important environmental factors correlated with an increased occurrence of dead flies included the abundance of dew on the vegetation during the night (dew point 4–21°C), and precipitation on the day before a collection (**Fig. 1**). Though results were not statistically significant, we observed a weak positive correlation between the number of dead flies and relative humidity, average daily temperature and dew points (r = 0.33, 0.36 and 0.33 respectively).

The two host species studied in Durham belong to the fly genera *Delia* (Anthomyiidae) and *Coenosia* (Muscidae) [18]. In spite of an abundance of other fly species (e.g., hoverflies and houseflies) at the research site, field site, we did not observe other fly families infected by *E. muscae*. We did not observe any difference between the two fly species regarding weather, localization of dead flies or their attachment to the plants except for the predominance of *Delia* cadavers in early spring and *Coenosia* cadavers in late spring to summer (**Fig. 1**).

### Micromorphology

Because initial attempts to isolate *E. muscae* into pure culture and to infect healthy flies in the laboratory were unsuccessful, observations of fungal development were limited to field-collected mycosed cadavers. Fungal infections produced similar symptoms in both fly hosts, which are typical for those described for *E. muscae* and related fly-infecting species (**Fig. 2** **–** **4**, **Text S1**) [6]. We compared the size of conidia (length and width) and number of nuclei in conidia found in *Delia* and *Coenosia*, traits which are essential for the identification of *Entomophthora* species [1], [6]. No significant differences in these characteristics between these species were observed at the p-value threshold >0.05 (p-values 0.086, 0.082 and 0.166 respectively).

**Figure 3:**
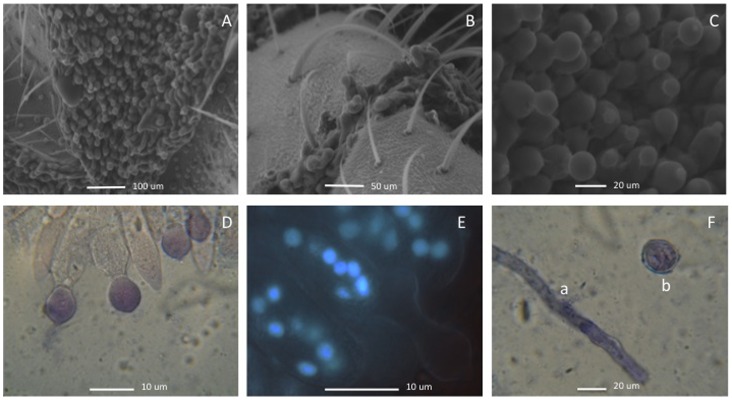
Conidiophores and hymenial layer of *Entomophthora*-infected flies from Durham, North Carolina, USA. A. Conidiophore layer between sclerites of fly cuticle, SEM. B. Conidiophores breaking through the cuticle of the fly, SEM. C. Conidiophore layer with young conidia, SEM. D. Conidiophores with young conidia stained with cotton blue. E. Nuclei in conidiophores stained with DAPI. F. Hypha (a) and young zygospore stained with cotton blue (b).

**Figure 4:**
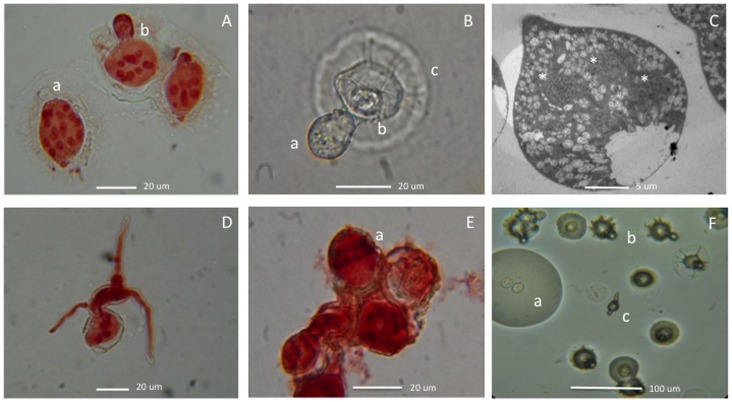
Vegetative and reproductive structures of *Entomophthora*-infected flies from Durham, North Carolina, USA. A. Plurinucleate conidium (a) and germinating conidium (b), stained with aceto-orcein. B. Secondary conidium (a), primary conidium (b), cytoplasm droplet (c). C. Nuclei (*) in conidium, TEM. D. Conidium germinating in water with mycelium; stained with aceto-orcein. E. Belt-like zone of nuclei in secondary conidium (a), stained with aceto-orcein. F. Conidia in water droplet (a), conidia with radially folded cytoplasm droplet (b), germinating conidium (c).

### Molecular Identification

Molecular identification with a fragment of the cytochrome c oxidase subunit I gene (COI) confirmed the identities of the flies from Durham, NC, as *Delia radicum* (NC State University Entomology database, GenBank accession number KC40476) and *Coenosia tigrina* (GenBank, accession numbers FJ025606, KC404075).

**Figure 5:**
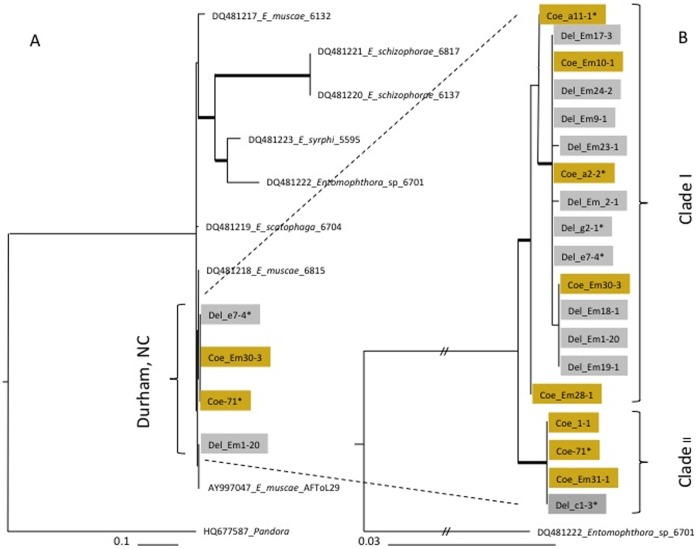
Phylogenetic analysis of *E. muscae* from Durham, NC, USA. Del_ indicate fungal sequences extracted from fly *Delia* (grey rectangles), Coe_ – from *Coenosia* (yellowish rectangles). Number after each sample (−71) indicates the number of identical sequences. Thick lines indicate branches with strong bootstrap support (>70%). A. ML tree of ITS sequences of genus *Entomophthora* with dominate genotypes from Durham, NC. B. ML tree of 120 ITS sequences of *Entomophthora*, isolated from *Coenosia* and *Delia* flies, representing all ITS sequences which differ in one or more bp positions or by host.

**Figure 6:**
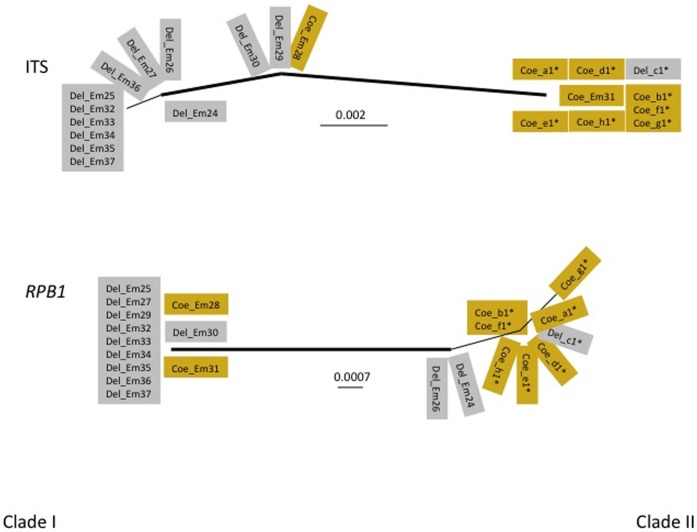
Unrooted trees of 22 randomly selected *E. muscae* samples using ITS and *RPB1* genes. Del_ indicate fungal sequences extracted from *Delia* (grey rectangles), Coe_ – from *Coenosia* (yellowish rectangles). Thick lines indicate branches with strong bootstrap support (>70%).

We identified our fungal samples as *E. muscae* using both traditional (morphologically based) keys and molecular evidence. The entire ITS1+ ITS2 region of *Entomophthora* is unusually large in comparison to other fungi: ∼ 1600 bp vs. 600–800 bp. BLAST searches resulted in 99% coverage and similarity to rDNA ITS1–5.8S-ITS2 of *E. muscae* AFToL-ID29 (**Table 1**). Phylogenetic analysis of the ITS region placed *E. muscae* samples from North Carolina together with *E. scatophagae* and *E. muscae* AFToL-ID29 within the genus *Entomophthora* (**Fig. 5** **A**). Using the ITS region, we determined the variability across the *E. muscae* populations from Durham. Seven haplotypes were found among 120 sampled ITS sequences (**Fig. 5** **B**), that grouped phylogenetically into clades I and II (44 and 76 samples, respectively) with significant statistical support. The clade I sequences were obtained mostly from *Delia* hosts, and clade II sequences were obtained mostly from *Coenosia*. Clade I includes the majority of samples collected from the second half of March through the first half of April (over 80% of the samples in this clade). Clade II is represented by samples collected mostly after mid-April (**Fig. 1**). Each fungal clade also contain fungal samples infecting the alternate fly species, including eight *Coenosia* isolates in the clade I, and three *Delia* isolates in the clade II. Despite a larger sample size of clade II, it is more phylogenetically uniform than is clade I (**Fig. 6**).

**Table 1:** Percentage of rDNA identity and gap lengths for gene fragments from *E. muscae* specimens from Durham compared to other published *Entomophthora* and outgroup species sequences.

	28S	ITS1–5.8S-ITS2	ITS1	5.8S-ITS2
*Entomophthora muscae*, Durham	KC404073	KC404074	KC404069*	KC404089**
*E. muscae* ***	**99–100/0**: EF392408, DQ273772, DQ481224–25 NG_027647	97–99/0–1: AY997047	99/0: GQ285865–66	98–99/0: DQ481217–18
*E. scatophagae*	99/0: DQ481226	–	99/0:GQ285867	97/2: DQ481219
*E. ferdinandii*	99/0: GQ285882	–	97–98/0: GQ285860–61	–
*E. schizophorae*	94/2: GQ285883,DQ481227–28	–	90–91/2: GQ285868–70	90–91/2: DQ481220–21,
*E. syrphi*	96/1: DQ481230	–	85/4:GQ285872, DQ481230	95/1: DQ481223
*E. planchoniana*	90/2: GQ285878	–	60/3:GQ285856	–
*Entomophthora* sp. ARSEF 6701	96/1: DQ481229	–	–	89/1: DQ481222
*E. grandis*	–	–	81/10: GQ285863	–
*E. chromaphidis*	–	–	40/4: GQ285848	–
*Entomophaga aulicae*	84/8: EF392372	–	89/2: U35394	89/2: DQ534746, DQ534753
*Pandora neoaphidis*	45/7: EF392405	93/2: HQ677587	–	–

99–100/0 - here 99–100% of identity and/0% of gaps to the whole length of fragment, bp.

* also includes genotypes KC404070–71, KC404078–88.

** also includes genotypes KC404090–103.

*** variability in different isolates.

Phylogenetic analyses of three additional unlinked genetic markers (*RPB1*, *RPB2*, and *EFL* genes) using randomly selected subsets of samples always divided the population into basically the same two major clades. Phylogenetic incongruence could be observed for every gene, however, suggesting that recombination is also occurring between populations (**Fig. 6** **–** **7**).

**Figure 7:**
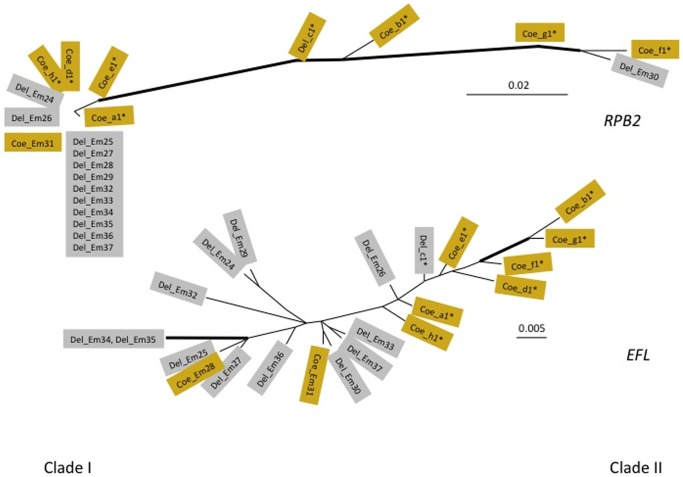
Unrooted trees of 22 randomly selected *E. muscae* samples using *RPB2* and *EFL*. Del_ indicate fungal sequences extracted from *Delia* (grey rectangles), Coe_ – from *Coenosia* (yellowish rectangles). Thick lines indicate branches with strong bootstrap support (>70%).

We used model-based clustering and algorithmic methods [19]–[22] to test whether populations of *E. muscae* were subdivided, to infer population parameters, to assign collected samples to subpopulations and to estimate gene exchange between them. Gene diversity was 0.77±0.06 for ITS (ten nucleotide positions in 809 bp of total fragment length, seven haplotypes); 0.67±0.11 (five in 744 bp, five haplotypes) for *RPB1*; 0.99±0.09 (three in 711 bp, 15 haplotypes) for *RPB2*; and 0.98±0.03 (17 in 504 bp, 14 haplotypes) for *EFL*. We tested the possibility that our sample represents one, two, three or four subdivided populations (**Table S1**). The highest likelihood values were obtained for two and three-population (Mean LnP −1336.22 and −1179.09, respectively), while these values were lower for one and four populations (−1822.37 and −1599.25, respectively). However, δ K for two populations was much higher than for three populations (i.e., 21.38 and 3.21, respectively). Phylogenetic analyses of each gene marker strongly supported two major lineages for every gene except *EFL* (100% for ITS and *RPB1*, 86% for *RPB2*, **Figs 6** **–** **7**). Several samples (∼5–25% or 1–6 out of 22) were not placed together with their clade for at least one marker. Fst values for each gene were: ITS - 0.43442, RPB1–0.29725, RPB2–0.62003, EFL - 0.22658. Thus, for these four loci 23 to 62% of the total genetic variation is distributed among subpopulations, with 38–77% of the variation within subpopulations. These values support phylogenetic evidence of recombination for each locus.

Evidence for two genetic subgroups within our *E. muscae* population is consistent with predominant patterns of host species utilization, and collecting season. Thus our set of *E. muscae* samples represents two morphologically identical but genetically distinct groups with some limited gene flow between them.

## Discussion

### Influence of Environment

Several observations suggest a connection between environmental factors and infection of flies by *E. muscae*. These include air temperature, relative humidity and dew point temperature. Relative humidity and temperature also affected fungal conidial discharge and germination rates [17]; both of these environmental parameters are linked indirectly to pathogenicity. However, the degree of this correlation could not be estimated more precisely because our analysis did not include many other important biological, environmental and anthropogenic factors such as the abundance of the hosts, their reproductive cycles, nutrition and behavior, their species diversity, the water-holding capacity of the habitat, or such disturbances as the mowing and watering of lawns. It is interesting that the highest numbers of dead flies were found when the minimum daily temperature and dew point were nearly equal (within ca. 2°C of each other). These conditions assure the largest amount of free water being generated in the environment. This free water may aid in the discharge of conidia, and encourage the fungus to proceed with conidial germination and infection. The absence of *Delia* flies in the late April through May collections is most likely explained by their aestivation when daytime temperatures exceed 22°C [23]. It is unclear why *Coenosia tigrina* flies were absent in our sampling early in spring. However the temperatures below 20°C inhibit the hunting activity in the closely related species *C. attentuata* [24]. Also, the developmental processes in *C. tigrina* (egg hatching, larval development, duration of the pupal stage) run twice as fast at 25°C as at 15°C [25]. Thus, there were more active *Coenosia* flies available for *Entomophthora* toward the end of spring whereas *Delia* flies were practically absent in the ecosystem. There is only a short period of time when both fly species life cycles overlap and contact between them–and possible cross-infections–might occur. The infection conditions and host availabilities effectively seem to separate these two populations of *Entomophthora*.

### Taxonomic Position

The North Carolina fungi studied here resemble both *E. muscae* and *E. scatophagae* in their biology, morphology and molecular characteristics. It is unknown whether our fungal pathovars could infect hoverflies (Syrphidae) or houseflies (Muscidae) but they did kill two unrelated species of flies from the families Anthomyiidae and Muscidae inhabiting the same environment. *Entomophthora muscae* is known to infect flies in all of these families [26]. Conidia of our samples are generally smaller than those typical of *E. scatophagae*. Conidial length and diameter are also smaller than in some of the *E. muscae* pathovars found on *Delia* and *Coenosia* flies by other authors: [6] (**Table 2**): 21–18 vs. 26–21 um, but are still within the range for *E. muscae* conidia. The few resting spores we observed were also much smaller than those typical of *E. muscae*: 15–18 vs. 30–40 µm diam. [27], respectively. However, insufficient numbers of spores were observed to allow meaningful evaluation of their sizes or their role in the *Entomophthora* life cycle. The BLAST results for the 28S and ITS fragment sequences and their resultant phylogenetic trees suggested our samples to be very closely related to *E. muscae*, while the phylogenetic analysis placed previously deposited sequences attributed to *E. scatophagae* and two of *E. muscae* isolates among those of our samples. However, *E. scatophagae* distinctively differs from our samples by the average number of nuclei per conidium: 17 vs. 12, respectively [28]; there are only two incomplete ITS sequences of *E. scatophagae* in GenBank available for genomic comparisons (**Table 1**). Our samples also differed from *E. schizophorae* (with 4–7 nuclei/conidium) infecting onion flies (*Delia antiqua*) in Europe [3], [29], [30].

**Table 2:** Summary of reported primary conidial sizes and numbers of nuclei per conidium (mean ± SE and range of means) of *E. muscae* isolates from different hosts and in species of the genus *Entomophthora* [4]–[6], [26], [29], [47].

Species	Number of nuclei per conidium	Length (um)	Diameter (um)
//*Delia*	16.4±0.3 [13.8–18.5]	26.9±0.5 [25.3–29.1]	21.4±0.5 [20.8–23.7]
//*Coenosia*	14.6±0.8 [14.5–14.9]	25.0±0.9 [24.3–25.7]	20.2±0.7 [19.3–21.1]
**//** ***Delia*** ** Durham**	**11.4±0.19 [9.5–13.4]**	**21.3±2.2 [19.1–23.5]**	**17.7±1.6 [16.1–19.3]**
**//** ***Coenosia*** ** Durham**	**11.8±0.21 [11.3–12.2]**	**21.7±1.6 [20.1–23.3]**	**17.7±1.3 [16.4–19.0]**
//*Leptohylemyia*	–	22.6±1.3	17.7±1.2
//*Drosophila*	–	19	16
//*Pollenia*	–	23	18.5
//*Lucilia*	–	16	13
//*Sarcophaga*	–	22 [15]–[39]	16 [11]–[28]
//*Syrphus*	–	25–29	21–23
//*Melanostoma*	–	29.8±1.1	22.8±1.5
//*Platycheirus*	–	28.6±1.4	23.4±1.1
//*Pegoplata*	13.0±1.12	27.4±1.23	21.4±0.94
//*Musca*	18.4±0.2 [16.1–20.7]	29.0±0.2 [25.2–31.5]	23.1±0.2 [21.1–25.2]
//*Scatophaga*	–	27.4±1.5	21.3±0.4
*E. muscae* s.l.	10–22 [6]–[30]	25 [13]–[40]	19.5 [11]–[30]
*E. scatophagae*	17 [12]–[19]	30 [27]–[35]	24 [21]–[25]
*E. culicis*	2	9–16	8–15
*E. erupta*	3–4	17–23	15–18
*E. planchoniana*	4–6	11–23	10–20
*E. schizophorae*	6±1 [4]–[10]	22.2±1.8 [17]–[25]	16.9±1.4 [12]–[22]
*E. syrphi*	16–24	27.5–31	22–24
*E. chromaphidis*	4–6	11–15	–
*E. terrestris*	–	13.3–18.9	8.3–9.7
*E. weberi*	6	12–18	11–16

//indicates morphological characteristics of collections from *E. muscae* species complex affecting these different muscoid fly hosts.

We were not able to resolve the relationships between *E. muscae*, *E. scatophagae* and our samples using the 28S and ITS regions of rDNA with any meaningful statistical support, but these sequences do unambiguously place our samples within the *E. muscae* species complex. Further definition of the relationships within the *E. muscae* species complex and the host specificities of its constituent taxa would benefit from more comprehensive molecular evaluation with additional markers. Detailed phylogenetic studies including more sequences and molecular markers would enable a more precise resolution of the position of the samples of *E. muscae* from North Carolina within the genus *Entomophthora*, and should help generally to resolve the *E. muscae* species complex.

### Fungal Population Structure and Evidence for Genetic Recombination

Using the ITS region sequences we found that the *E. muscae* populations in Durham were not uniform and consisted of several haplotypes grouped into two well supported clades. The majority of ITS sequences obtained from *Delia* flies were distinct from those from *Coenosia*, as evidenced by the ten bp difference in the ca. 800 bp fragment of ITS rDNA spacer region). This genetic difference is also associated with the greater affinity of both clades I and II for one fly host species over another. This division into two clades is also well supported by phylogenetic and population structure analysis of additional gene markers. We have also found “*Delia*-type” ITS in fungal DNAs isolated from *Coenosia* flies and *vice versa*. Infection of several hosts from the same family Muscidae by one *E. muscae* population is possible in nature, although laboratory experiments have shown considerable host specificity and decrease of infection efficiency for less closely related hosts [10], [31].

Despite their smaller size, *Coenosia* flies prey upon *Delia* flies in nature, and both flies were infected by the *E. muscae* group in Michigan, USA ([16], Carruthers, personal communication). Our observations of frequent physical contact between these two species at the observation sites (*Coenosia* may attempt–even if unsuccessfully–to attack larger flies or to land on fly cadavers) and abundant conidial “halos” around dead flies on the plants makes infection of *Coenosia* by secondary conidia possible. *Entomophthora* representatives of both clades I and II were able to infect both hosts, although with different efficiency. This resulted in different numbers of collected dead *Coenosia* and *Delia* flies infected with pathovars from clades I and II.

Using host infection prevalence, molecular data and phylogenetic analysis, we present the first direct evidence of a complex structure of *E. muscae* populations over the course of a single season. Differences in the ITS, *RPB1*, *RPB2* and *ELF* haplotypes allow us to propose the existence of separate subpopulations of the *E. muscae* population inhabiting the Durham area of North Carolina, USA. These two subpopulations may represent cryptic species that infect different hosts. Samples of clade II could represent an emerging new species from a broader genetic “landscape” of ancestral haplotypes, yet are incompletely separated. However, the pathogens’ ability to infect other hosts, the presence of intermediate haplotypes or hybrids (Coe_a11, Coe_Em28, Coe_Em31, Del_Em26), and phylogenetic evidence of recombination suggest that the speciation process is not completed. Some genetic recombination might still occur between these two clades, although the details for sexual processes in these species remain unknown. Perhaps sex occurs in rare resting spores, which were observed in *Coenosia*; during the epizootic period these fungi reproduce clonally. All entomophthoralean fungi have been considered up to now strictly clonal and homothallic [32]. Our finding can also expand our perception of sexual reproduction and species concepts in the *E. muscae* species complex. Each population of *E. muscae* in certain locations might consist of several genotypes with limited ability to cross, which can serve as another explanation of the observed phenomenon. Host utilization by *E. muscae* at each location might vary from year to year depending on weather, host availability, and presence and abundance of available pathogen genotypes in its populations. This statement can be supported by our one-day observation of *E. muscae* infection in *Delia*, *Coenosia* and *Scatophaga* flies on a small wheat field, made in April 2012 in Swannanoa valley near Asheville, NC (Gryganskyi et al., unpublished). Together with the presumed ability of “*Delia*” haplotypes to infect *Coenosia* flies and *vice versa*, and their posited genetic recombination, it appears that the *E. muscae* species complex can utilize several hosts consecutively during the same infection season, thereby increasing the overall fitness of its populations.

### Conclusions

Populations of *Entomophthora muscae* in NC consist of several ITS haplotypes infecting different hosts from at least two different fly families: *Delia radicum* (Anthomyiidae) and *Coenosia tigrina* (Muscidae). Two distinct dominating ITS haplotypes (clades I and II) are common and coexist mainly in the respective hosts during the same infection period, along with other rarer haplotypes. These haplotypes can be recognized as cryptic species, with their genetic separation possibly reinforced by shifts to different host species. Each of these cryptic species predominates at a different time, in relation to host abundance. Other ITS haplotypes are rare, but do occur and might represent hybrids between the dominant fungal lineages. The ability of each fungal species to infect other hosts naturally is limited, but we have shown that it does occur. Predation by *Coenosia* flies on *Delia* might serve as a conduit for genetic exchange between the two fungal clades. Incongruences between phylogenetic clades I and II based on four loci-ITS, *EFL, RPB1* and *RPB2*-might constitute evidence for some degree of genetic recombination between *Entomophthora* populations. Our results can also serve as the first evidence of sexual recombination in *Entomophthoromycota*, which have heretofore been considered to be homothallic and purely clonal zygomycetes.

## Materials and Methods

### Sampling

Infected flies were collected during spring 2011–2012 in the city of Durham, North Carolina (USA), north of Duke University’s West Campus. Study sites were five community lawns located in several places along Erwin Road, and these were sampled between 8 and 10 am every day, except during heavy rains. All collection areas are freely accessible for public use; no special permits and approvals were required. All sampling sites, areas of ca. 100 m^2^ each, were watered and mowed weekly during the observation period. At each visit the lawn was examined by walking in a grid pattern, removing dead flies, which could be seen at the tips of the lawn plants. All fly specimens were collected post-mortem, and these flies and their pathogens do not belong to protected, rare or endangered species. We removed most infected flies for identification purposes; 3–4 infected fly cadavers were left in the place to observe the duration of their attachment to a plant. Thus dead flies could serve as an infection source for only a short period of time. City weather data were downloaded from RDU Weather Reports [33].

### DNA Extraction and Amplification

DNA from infected flies was extracted using a CTAB/chloroform extraction technique [34]. Polymerase chain reactions (PCR) to identify both the fungi and their dipteran hosts were performed with the primers EmITS1 and EmITS4 for ITS region [35], LROR and LR3 for 28S rDNA [36], [37] for the fungi, and the mitochondrial cytochrome c oxidase (CO I) [38] for each fly sample. For additional analyses of structure and genetic recombination within *Entomophthora* populations we used primers for the domains 5–6 of *EFL* gene: Emusc_aEFL_f CGGAGATATCCAGGGACAGA and Emusc_aEFL_r GGAGGGAAGCTCAACGAAGT; and 6^th^ domain of *RPB1* gene: Emusc_RPB1_IIf GTCCCGAATGGATGGTTTTA and Emusc_RPB1_IIr AGGGGACACAATCTGCTTTG; and 5^th^ domain of *RPB2* gene: Emusc_RPB2_IIf GCATGGAGTTTCTTCGCTTC and Emusc_RPB2_IIr ACCACCATCTCGAGAACGAC. All PCR reactions were performed using Apex *Taq* DNA polymerase (Genesee Scientific, San Diego, CA, USA), purified and sequenced using previously published protocols [39]. Data and alignments were submitted to GenBank (**Table 1**) and TreeBASE (http://purl.org/phylo/treebase/phylows/study/TB2:S12923). We have used 120 ITS sequences as the main markers for the Durham populations. Additionally, we have used partial sequences of *EFL*, *RPB1* and *RPB2* (22 sequences for each gene) as additional markers for evaluating the diversity and structure of the *Entomophthora* populations. For the last analysis we randomly selected three rows (24 total excluding two bad quality DNA samples) from two 96 well plates with *Entomophthora* DNAs.

### Identification

ITS regions were used for fungal identification and species matched via BLAST similarity to available GenBank sequences of *Entomophthora* sp. A phylogenetic analysis incorporating available internal transcribed spacer data in GenBank was performed which included 120 sequences for the whole ITS region. The fungus was also identified using morphological keys [6], [26], [40]. Flies were identified to the genus level using morphological keys [18], [41] and were further categorized molecularly using COI primers, sequencing, and BLAST analysis [38] (GenBank accession numbers KC404075, KC404076).

### Microscopy

Infected flies were placed on glass microscope slides to collect conidia for direct visual examination. Measurements of conidial size and numbers of conidial nuclei were performed in two sets, examining 100 conidia each from different samples collected in different months. Numbers of conidial nuclei were counted from conidia stained with aceto-orcein [42] or DAPI [43]. Scanning electron microscopy (SEM) and transmission electron microscopy (TEM) were conducted using FEI XL30 SEM-FEG and FEI Tecnai GI Twin microscopes, respectively, at the Duke Shared Materials Instrumentation Facility. Conidial sizes and nuclear numbers were checked morphologically throughout the whole infection period, at least twice a month. Observations on spore germination and subsequent development were attempted with conidia discharged from infected flies and grown on malt agar (VWR International, Bristol, CT, USA), Sabouraud dextrose agar [44], Grace’s insect tissue culture medium with 5% fetal bovine serum (Sigma-Aldrich Co., St. Louis, MO), and humid chambers [45].

### Analysis of Environment Influence and *E. muscae* Population Structure

The influence of environmental factors on fly infection was estimated by calculating the linear correlation coefficient in STATISTICA 8.0 (Statsoft, Tulsa, OK). Phylogenetic tree congruency for different loci was estimated visually. Fungal clades were determined using Structure 2.3.4 [19]. Main population structure parameters were identified using IMa [21], [22] and Arlequin 3.5 [46].

The materials are preserved in the collections of Duke Herbarium (NC, USA), the ARS Collection of Entomopathogenic Fungal Cultures (USDA-ARS, NY, USA) and the Jena Microbial Resource Collection (JMRC, Germany). DNA samples were deposited at Duke University and JMRC.

## Supporting Information

Table S1 **Mean Likelihood values, their standard deviation and δ K for one, two, three and four populations (K) hypotheses** [**19]**, [47] **.**
(DOCX)

Text S1 **Morphological description of fungal infection in the flies** ***Delia radicum*** **and** ***Coenosia tigrina.***
(DOCX)
