# Supplementary material for: Sequential Utilization of Hosts from Different Fly Families by Genetically Distinct, Sympatric Populations within the Entomophthora muscae Species Complex
Source: PLoS One. 2013 Aug 8;8(8):e71168. doi: 10.1371/journal.pone.0071168 (PMC3738597; doi:10.1371/journal.pone.0071168)
Supplement: Table S1 — Mean Likelihood values, their standard deviation and δ K for one, two, three and four populations (K) hypotheses [19] , [47] . (DOCX) [file pone.0071168.s001.docx]

**Supplementary Table 1.** Mean Likelihood values, their standard deviation and δ K for one, two, three and four populations (K) hypotheses [[19](#_ENREF_19),[47](#_ENREF_47)].

| **K** | **Reps** | **Mean LnP(K)** | **Stdev LnP(K)** | **Ln'(K)** | **\|Ln''(K)\|** | δ **K** |
| --- | --- | --- | --- | --- | --- | --- |
| 1 | 11 | -1822.336364 | 2.296638 | — | — | — |
| 2 | 10 | -1336.220000 | 15.384465 | 486.116364 | 328.986364 | 21.384322 |
| 3 | 10 | -1179.090000 | 179.837602 | 157.130000 | 577.290000 | 3.210063 |
| 4 | 10 | -1599.250000 | 618.193830 | -420.160000 | — | — |
