# Supplementary material for: Sequential Utilization of Hosts from Different Fly Families by Genetically Distinct, Sympatric Populations within the Entomophthora muscae Species Complex
Source: PLoS One. 2013 Aug 8;8(8):e71168. doi: 10.1371/journal.pone.0071168 (PMC3738597; doi:10.1371/journal.pone.0071168)
Supplement: Text S1 — Morphological description of fungal infection in the flies Delia radicum and Coenosia tigrina. (DOCX) [file pone.0071168.s002.docx]

**Supplementary Text 1.**

**Morphological description of fungal infection in the flies *Delia radicum* and *Coenosia tigrina***

Major growth of the fungus occurred on the cadaver by covering the abdomen. It became a dense layer of sporulating fungal mass, which almost completely coalesced on the ventral side, leaving only 3-4 cuticular segments (tergites) intact. A few tufts of conidiophores emerged from the joints of the legs and other weak points of the cuticle

(**Fig. 2 B, E; 3 A, B**). We observed numerous, simple, club-shaped multinucleate conidiophores 75-120 μm long and 22-27 μm wide (**Fig. 3 C-E**). The nature of the cuticular damage suggested that conidiophores exited the host body mainly by mechanical rupture rather than by enzymatic activity (**Fig. 2 E**). The hymenial layer of conidiophores developed between and eventually covered the dorsal tergites and the ventral pleuron of the fly’s abdomen (**Fig. 2 B, E**). This mass of conidiophores was grayish white or a pale color in the early morning, and turned pinkish brown, collapsed and desiccated later in the day. We did not observe any rhizoids. Conidiophores produced single broadly ellipsoidal (campanulate or bell-shaped) hyaline conidia with a prominent apical point and broad, flat, usually slightly convex papilla, 12-40 x 10-30 um, usually 18-25 x 18-20 μm (**Fig. 4 A-C, E, F**). The maximum length:width ratio for the conidia was 1.1-1.4, but mostly ranged between 1.16-1.3. Conidia contained a maximum of 10-13 nuclei, but averaged 12 (**Table 2**). Secondary conidia often contained a large central vacuole.

Conidia were forcibly ejected to a distance of up to 3 cm and formed a powdery “cloud” around the infected fly on the substrate or on the lid of Petri dish (**Fig. 2 C**). Discharged primary conidia were usually seen to be embedded in a “halo” of protoplasm or other material discharged along with the conidium and that became radially folded as it dried out (**Fig. 4 B, F**). Further development of the primary conidia started within an hour of discharge, usually by the formation of a secondary conidium (**Fig. 4 A, B**). Secondary conidia resembled primary ones, but were smaller, broadly obovoid, and without any apparent apiculus. Rarely, initiation of a germ tube occurred, which could grow to a length 100-800 um, but germ tubes usually collapsed the same or the following day (**Fig. 4 D**).

Hyphal bodies were short, rod-like, protoplastic, multinucleate, and filled with numerous oil droplets (**Fig. 3 E**). We have found very few immature resting spores in the dissections of infected *Coenosia* flies. These resting spores were spherical, 30-40 μm in diameter, with smooth-surfaced cell walls 2.5-3.5 μm thick, and occasionally showed remnants of the parental cell(s) from which they formed (**Fig. 3 F**). We could not determine whether these resting spores formed as zygospores (from gametangial conjugations) or azygospores (without any prior conjugation) [[6](#_ENREF_6)].

Nuclei contained more or less central nucleoli, 0.74–1 x 1–1.75 um, and prominent granules of condensed chromatin during interphase that stained readily with aceto-orcein or DAPI. Nuclei were ovoid to subglobose, 2-4x2-6.5 μm (mostly 3-4x4-5 μm) and either were distributed throughout the length of the cells (**Fig. 3 E; 4 A**) or often, in secondary conidia, in two median “belts” in the widest part of the conidium (**Fig. E**). All cellular structures had numerous vacuoles and/or oil droplets of differing sizes (**Fig. 4 B-D**). Septa occurred in fewer than 1% in all hyphal bodies.
